# Contrasting pre- and post-COVID changes in within-host *gp60* diversity among human-infecting *Cryptosporidium* species

**DOI:** 10.1098/rsos.261952

**Published:** 2026-11-11

**Authors:** Juan Carlos Garcia-R, Jack Cameron, Matthew C. Knox, David T. S. Hayman

**Affiliations:** Massey University, School of Veterinary Sciences, Palmerston North, New Zealand

**Keywords:** COVID-19, *Cryptosporidium* diversity, metabarcoding, transmission ecology and dynamics

## Abstract

Population connectivity influences genetic diversity by facilitating exchange among populations. For enteric parasites, connectivity depends on host contact and environmentally mediated transmission, suggesting that disruptions to human movement could reduce diversity within infections. The COVID-19 pandemic provided a natural experiment to test this prediction. We used high-throughput *gp60* metabarcoding to compare within-host amplicon sequence variant (ASVs) richness, diversity and composition before and after the pandemic in three human-infecting *Cryptosporidium* species with contrasting transmission ecologies: anthroponotic *Cryptosporidium hominis*, zoonotic *Cryptosporidium parvum* and wildlife-associated *Cryptosporidium cuniculus*. Primary analyses excluded 26 epidemiologically linked *C. hominis* infections from a 2023 outbreak; sensitivity analyses included these samples. Post-COVID, *C. hominis* showed significantly lower Shannon, Simpson and inverse-Simpson diversity, 63% fewer detectable ASVs and altered ASV composition. *Cryptosporidium parvum* showed compositional differences but no significant reduction in diversity or richness, whereas *C. cuniculus* showed no clear temporal change. Including outbreak-associated *C. hominis* samples attenuated diversity reductions but retained significant declines in Shannon diversity, Simpson diversity, ASV richness and composition. These findings are consistent with reduced parasite connectivity following disruption of predominantly human-associated transmission and indicate that major changes in transmission conditions can rapidly reshape the genetic diversity maintained within parasite infections.

## 1. Introduction

Genetic diversity within populations is shaped by a dynamic interplay of mutation, genetic drift, selection, recombination and gene flow [1]. A central prediction of population genetic theory is that reductions in connectivity among populations limit the movement of genetic variants, increase the effects of drift and ultimately reduce genetic diversity [2,3]. This principle forms the foundation of modern molecular ecology, where processes such as habitat fragmentation, dispersal barriers and demographic bottlenecks are recognized for their impact on genetic variation across a wide range of taxa [4–8]. However, while the consequences of geographic isolation and fragmentation have been widely studied in free-living organisms, opportunities to examine the evolutionary effects of abrupt reductions in transmission of parasitic microorganisms, where transmission networks represent the primary mechanism connecting populations, are rare [9–12].

For parasites transmitted through faecal-oral pathways, transmission connects parasite populations through both host-to-host contact and environmentally mediated routes. Each infected host can be considered a habitat patch occupied by a local parasite population, where transmission moves parasite genotypes among hosts directly or through contaminated water, food, surfaces and other environmental media. The structure and connectivity of these transmission networks play a crucial role in parasite evolution. Factors such as host movement, contact frequency, sanitation, environmental contamination and exposure to shared water or food sources collectively influence the exchange of parasite genotypes among infections, thereby affecting population size, genetic variation and adaptive evolution [13–15]. In this context, the arrangement of host contact networks determines the degree of connectivity among parasite populations and regulates the exchange of genetic variation across the broader metapopulation. Therefore, reductions in transmission opportunities can have consequences analogous to habitat fragmentation: they decrease parasite connectivity, limit the movement of genetic variants, reduce opportunities for the simultaneous transmission of genetically distinct lineages (co-transmission) and increase the influence of stochastic processes [16].

The effects of transmission disruption may be detectable not only at the population level but also within individual infections. Mixed infections arise when hosts are exposed to multiple parasite lineages through repeated transmission events or through genetically heterogeneous sources of infection [17,18]. The accumulation of multiple lineages within hosts contributes to within-host genetic diversity and provides opportunities for competition, recombination and the persistence of rare variants. When transmission networks contract, however, opportunities for acquiring genetically distinct lineages decline [19,20]. Under such conditions, infections are expected to become genetically simpler, characterized by fewer co-occurring variants and reduced within-host diversity. This prediction has received indirect support from studies of viral and bacterial pathogens, where reduced transmission has been associated with lineage loss, founder effects and transmission bottlenecks [21,22]. Empirical evidence for such effects remains rare for eukaryotic parasites, particularly those with complex transmission dynamics and substantial standing genetic diversity [23,24]. Thus, it is still unclear whether large-scale disruption of transmission networks can leave detectable signatures in the within-host genetic diversity of parasite populations.

The COVID-19 pandemic created a natural experiment for addressing this question. Non-pharmaceutical interventions, including lockdowns, travel restrictions, physical distancing measures and enhanced hygiene practices, substantially altered patterns of human contact and mobility worldwide [25,26]. These interventions reduced opportunities for transmission of numerous infectious agents and, in effect, generated a temporary fragmentation of human transmission networks [27,28]. From a population genetic perspective, the pandemic therefore represents a large-scale perturbation of parasite transmission connectivity, creating conditions analogous to demographic bottlenecks in natural populations. This situation provides a unique opportunity to investigate how abrupt reductions in host movement and contact influence parasite diversity.

*Cryptosporidium* species are particularly well suited to testing these processes because they differ markedly in their transmission ecology. *Cryptosporidium hominis* is transmitted predominantly between humans [29,30] and therefore depends largely on human-derived transmission pathways, including both person-to-person and environmentally mediated transmission originating from human infections. By contrast, *C. parvum* is maintained through both human and animal reservoirs [31,32], while *C. cuniculus* is primarily associated with wildlife hosts, with humans as comparatively incidental hosts [33]. These differences generate clear predictions regarding the consequences of transmission disruption. If human connectivity is a major determinant of parasite genetic diversity, then reductions in human-to-human transmission should disproportionately affect *C. hominis*, whereas species maintained in non-human reservoirs should be comparatively buffered against diversity loss. Furthermore, within-host genetic diversity in *Cryptosporidium* reflects the co-occurrence of genetically distinct parasite populations within individual infections, including multiple subtype families and variants within the same subtype [34–36].

Recent advances in sequencing technologies provide an opportunity to test these predictions directly by detecting subtle within-host changes in genetic composition. Traditional subtype-based approaches typically characterize infections using a single consensus sequence and therefore underestimate the complexity of mixed infections [34,36–40]. By contrast, metabarcoding resolves individual amplicon sequence variants (ASVs), unique DNA sequences differing by as little as a single nucleotide. This approach enables quantitative assessment of within-host diversity at a biologically relevant scale and the detection of low-frequency variants within infections that would otherwise remain hidden [41]. Because mixed infections and minor variants reflect the cumulative outcome of transmission processes, changes in within-host diversity may provide a sensitive indicator of altered connectivity within transmission networks [11]. Consequently, ASV richness and diversity can be viewed as ecological and evolutionary measures of the genetic complexity maintained within transmission networks.

Here, we use *gp60* metabarcoding to investigate whether the COVID-associated disruption of transmission opportunities was accompanied by changes in within-host genetic diversity and ASV composition in three human-infecting *Cryptosporidium* species. We hypothesized that transmission ecology would influence both the diversity maintained within infections and the response to changes in human contact patterns. Specifically, we predicted that the predominantly anthroponotic *C. hominis* would exhibit a stronger post-COVID reduction in ASV richness and diversity than the animal-associated species *C. parvum* and *C. cuniculus*, whose transmission can be maintained through non-human reservoirs. To focus on broader non-outbreak patterns, our primary analysis excluded a set of post-COVID *C. hominis* infections associated with a single geographically and temporally clustered outbreak. These outbreak-linked infections were then included in a sensitivity analysis to assess how their inclusion affected the observed temporal associations.

## 2. Materials and methods

### 2.1. Sample collection

Clinical specimens positive for *Cryptosporidium* were obtained through routine diagnostic surveillance and classified as pre-COVID (before March 2020) or post-COVID (after May 2020) according to collection date. Anonymous fresh faecal samples positive for cryptosporidiosis were collected from symptomatic humans in different regions of both the North and South Islands in New Zealand. *Cryptosporidium*-positive stool samples were sent from accredited diagnostic laboratories to the Hopkirk Research Institute at Massey University for molecular analyses under a Ministry of Health contract. Isolates were analysed through routine public health investigation activities.

### 2.2. Sequencing

DNA was extracted from approximately 200 mg of faecal material using the Zymo Quick-DNA Fecal/Soil Microbe kit (Irvine, CA, USA) supplemented with an additional bead-beating disruption step to enhance oocyst lysis, following the manufacturer's protocol. Extracted DNA was stored at −20°C until further analysis. PCR, library preparation and sequencing were conducted using a high-throughput *gp60* metabarcoding workflow previously published [34]. In brief, nested PCR was performed to amplify an approximately 400 bp fragment of the *gp60* gene using the primer set reported by Zahedi *et al.* [36] and Platinum Taq DNA polymerase (Invitrogen). The PCR cycling conditions consisted of an initial denaturation at 94°C for 3 minutes and then 40 cycles of 94°C for 45 s, 54°C for 45 s and 72°C for 1 minute, followed by a final extension step at 72°C for 7 minutes. Internal primers were modified to incorporate Illumina MiSeq adapter sequences at the 5′ end, following a two-step amplicon library preparation protocol. PCR contamination controls were used, including negative controls. PCR products were purified according to Illumina recommendations, quantified on a Qubit 4.0 instrument (Life Technologies), and normalized before pooling. Sequencing was performed on an Illumina MiSeq using 500-cycle v2 chemistry (2 × 250 bp paired-end reads). An Illumina PhiX library was spiked at 20% as an internal sequencing control. Raw sequence reads are deposited in the NCBI Sequence Read Archive under BioProject accession PRJNA1507456.

### 2.3. Analyses

Raw Illumina reads were processed within the Quantitative Insights Into Microbial Ecology 2 (QIIME 2) environment [42]. DADA2 [43] was used for filtering and trimming, dereplication, merging paired reads, removing chimeras and denoising to infer exact ASVs. DADA2 was implemented with the BANDSIZE parameter set to 2, following previous recommendations for *gp60* metabarcoding analyses, as this setting facilitates accurate retention and discrimination of individual trinucleotide repeat units within the highly polymorphic repeat region [44]. ASV count tables were imported into the R environment for downstream analysis and visualization. The final ASV table contained a mean of approximately 25 000 non-chimeric, quality-filtered reads per sample.

Within-sample diversity was quantified using ASV richness and the Shannon, Simpson and inverse Simpson diversity indices. Shannon, Simpson and inverse Simpson diversity were analysed using linear models containing COVID period, species, their interaction and log-transformed read depth as predictors. ASV richness was analysed using a negative-binomial generalized linear model with the same predictors. Estimated marginal means were used to calculate pre- to post-COVID changes within each species, differences in temporal change among species and pairwise comparisons among species within each period.

For beta-diversity analyses, ASV counts were transformed to relative abundances before Bray–Curtis dissimilarities were calculated. Distance matrices and principal coordinates analyses were calculated separately within each species. Temporal differences in ASV composition were tested using species-specific permutational multivariate analysis of variance (PERMANOVA) with 999 permutations. Homogeneity of multivariate dispersion was assessed using betadisper in the VEGAN R package [45–47] because significant PERMANOVA results can be influenced by differences in within-group dispersion. False-discovery-rate corrections were applied across the three species-specific PERMANOVA and dispersion tests. Binary Jaccard distances were analysed as a presence–absence sensitivity analysis. A pooled species × COVID PERMANOVA was additionally used as an omnibus test of whether temporal compositional changes differed among species.

The primary analyses excluded 26 post-COVID *C. hominis* infections associated with a 2023 outbreak in Queenstown. These epidemiologically linked infections were excluded to prevent a single geographically and temporally clustered transmission event from dominating estimates intended to represent broader non-outbreak patterns. All alpha- and beta-diversity analyses were then repeated with the outbreak-linked infections included as a sensitivity analysis.

## 3. Results

### 3.1. Alpha-diversity patterns

The primary dataset comprised 307 samples after exclusion of the 26 outbreak-linked post-COVID *C. hominis* infections. This included 116 *C. hominis* infections (100 pre-COVID and 16 post-COVID), 167 *C. parvum* infections (78 pre-COVID and 89 post-COVID) and 24 *C. cuniculus* infections (12 in each period). Within-host diversity declined markedly in non-outbreak *C. hominis* infections during the post-COVID period (figure 1). Adjusted Shannon diversity was 1.361 units lower post-COVID (95% CI: −1.768 to −0.954; *p* < 0.0001), Simpson diversity was 0.424 units lower (95% CI: −0.523 to −0.326; *p* < 0.0001) and inverse Simpson diversity was 2.185 units lower (95% CI: −3.940 to −0.430; *p* = 0.0149). Estimated post-COVID ASV richness was 0.37 times the pre-COVID richness (95% CI: 0.253–0.541; *p* < 0.0001), corresponding to an estimated 63% reduction.

**Figure 1.**
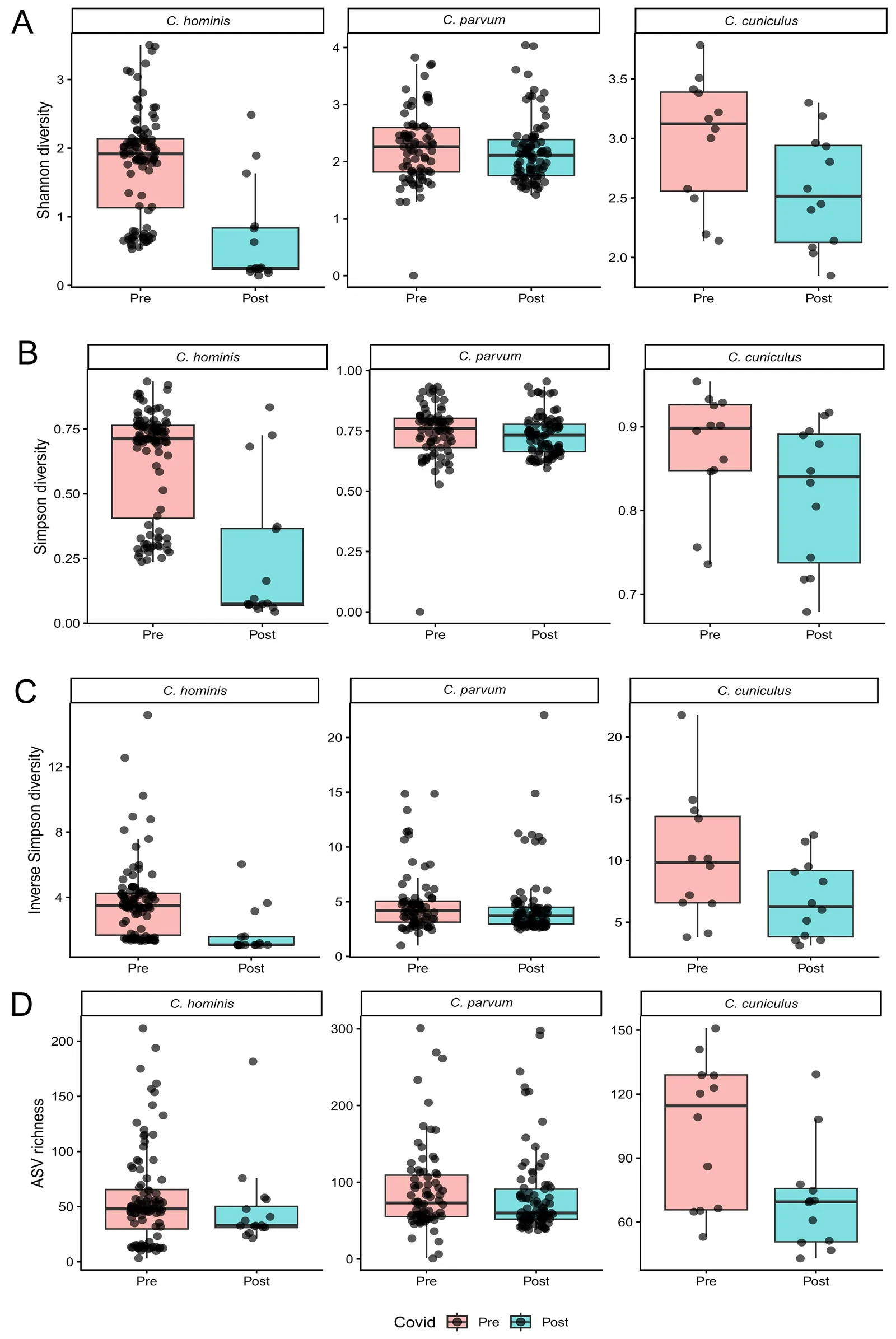
Comparison of within-host diversity between pre- and post-COVID samples. The figure excludes the 26 outbreak-linked post-COVID *C. hominis* infections. (A) Shannon diversity, (B) Simpson diversity, (C) inverse Simpson diversity and (D) amplicon sequence variant (ASV) richness for *C. hominis*, *C. parvum* and *C. cuniculus*. Boxes show the interquartile range with the median; whiskers represent 1.5 × IQR, and points represent individual samples.

By contrast, *C. parvum* showed no significant pre- to post-COVID change (figure 1) in Shannon diversity (estimate = −0.091, *p* = 0.3685), Simpson diversity (−0.014, *p* = 0.5759), inverse Simpson diversity (−0.419, *p* = 0.3370) or ASV richness (ratio = 0.907, *p* = 0.2732). For *C. cuniculus*, Shannon diversity (−0.435, *p* = 0.1025), Simpson diversity (−0.054, *p* = 0.4015) and ASV richness (ratio = 0.714, *p* = 0.1473) did not differ significantly between periods (figure 1). Inverse Simpson diversity was significantly lower post-COVID (estimate = −3.325, *p* = 0.0039). This isolated result, based on only 12 infections per period, was not consistently supported by the other diversity measures, which may reflect sampling variation among a small number of highly uneven infections rather than a genuine population-level signal.

The species × COVID interaction was significant for Shannon diversity (*p* < 0.0001), Simpson diversity (*p* < 0.0001), inverse Simpson diversity (*p* = 0.0212) and ASV richness (*p* = 0.0001), indicating that temporal changes differed among species. The reduction in *C. hominis* was significantly greater than that in *C. parvum* for Shannon diversity, Simpson diversity and ASV richness. The *C. hominis* reduction was also greater than that in *C. cuniculus* for Shannon and Simpson diversity, although their richness changes did not differ significantly after Tukey adjustment.

### 3.2. Beta-diversity patterns

Species-specific beta-diversity analyses also identified contrasting temporal patterns (figure 2). Relative-abundance Bray–Curtis composition differed significantly between periods for *C. hominis* (PERMANOVA *R*² = 0.132, *p* = 0.001, FDR-adjusted *q* = 0.0015) and *C. parvum* (*R*² = 0.098, *p* = 0.001, *q* = 0.0015), but not for *C. cuniculus* (*R*² = 0.041, *p* = 0.497). The pooled species × COVID interaction was significant (*R*² = 0.048, *p* = 0.001), indicating that the magnitude of temporal change differed among species. Bray–Curtis dispersion was lower post-COVID for both *C. hominis* and *C. parvum*. Mean distance to the group centroid declined from 0.550 to 0.381 for *C. hominis* and from 0.571 to 0.470 for *C. parvum*. The corresponding dispersion tests were significant after false-discovery-rate correction. The Bray–Curtis PERMANOVA results may therefore reflect both temporal differences in ASV profiles and changes in within-period heterogeneity. For *C. hominis*, however, the binary Jaccard analysis also detected a significant temporal difference (*R*² = 0.094, *p* = 0.001, *q* = 0.0015) without a significant dispersion difference (*p* = 0.811), providing stronger evidence of turnover in ASV presence or absence. For *C. parvum*, the Jaccard PERMANOVA was significant but remained accompanied by a dispersion difference, requiring more cautious interpretation. Neither the Jaccard composition nor dispersion differed significantly for *C. cuniculus*.

**Figure 2.**
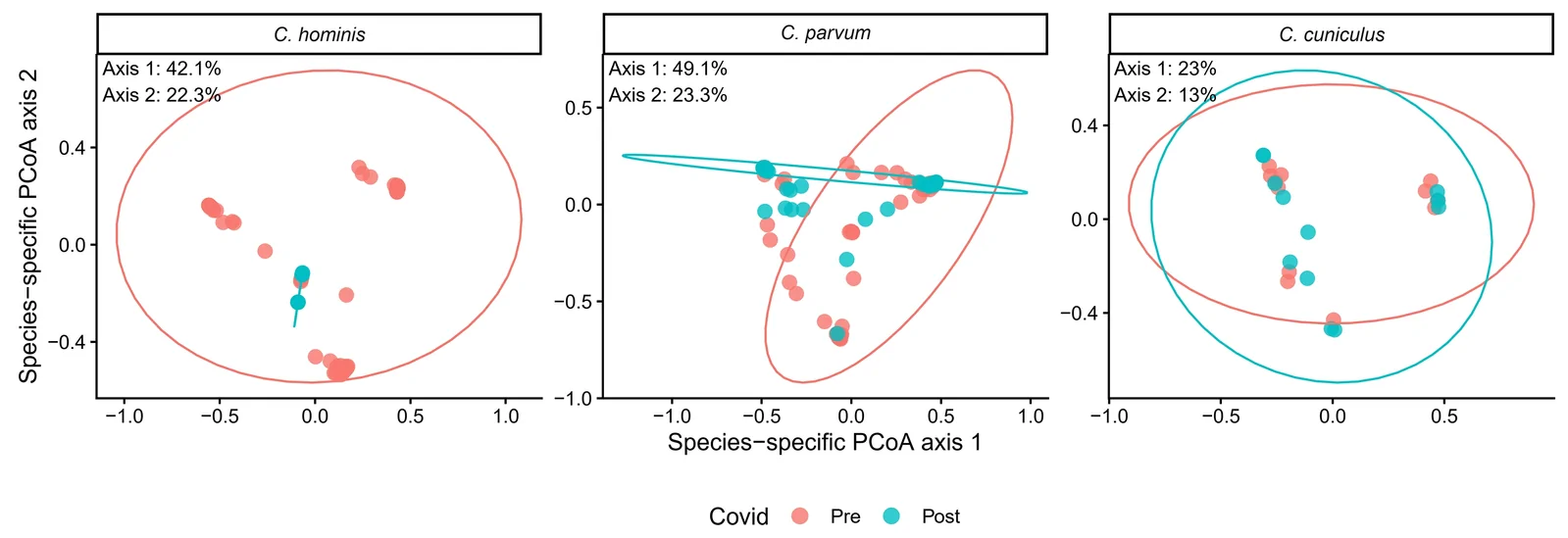
Principal coordinates analysis (PCoA) based on Bray–Curtis dissimilarity of *gp60* amplicon sequence variants. Samples are coloured according to the sampling period (pre- and post-COVID). Separate PCoAs were calculated within each species using Bray–Curtis dissimilarities derived from relative-abundance-transformed ASV profiles. Axis percentages and coordinates are specific to each species and are not directly comparable among panels. Temporal differences were tested using species-specific PERMANOVA, and homogeneity of multivariate dispersion was assessed using betadisper. The post-COVID *C. hominis* ellipse is unstable but included for completeness. Panels correspond to *C. hominis*, *C. parvum* and *C. cuniculus*.

### 3.3. Diversity patterns including outbreak-associated samples

Inclusion of the 26 outbreak-linked *C. hominis* infections increased the post-COVID *C. hominis* sample size from 16 to 42 and attenuated the estimated reductions in alpha diversity (supplementary material). Nevertheless, *C. hominis* retained significantly lower post-COVID Shannon diversity (estimate = −0.450, *p* = 0.0014), Simpson diversity (−0.089, *p* = 0.0137) and ASV richness (ratio = 0.530, *p* < 0.0001), corresponding to an estimated 47% reduction in richness. Inverse Simpson diversity was no longer significantly different (estimate = −0.735, *p* = 0.2019). The species × COVID interaction remained significant for richness but was attenuated below the conventional 0.05 threshold for Shannon, Simpson and inverse Simpson diversity. Inclusion of outbreak-linked infections did not remove the temporal difference in *C. hominis* ASV composition (supplementary material). The Bray–Curtis effect increased to *R*² = 0.212 (*p* = 0.001), and the binary Jaccard effect increased to *R*² = 0.150 (*p* = 0.001). The Jaccard dispersion difference remained non-significant (*p* = 0.623). Thus, the primary result was not dependent on the exclusion of the outbreak-linked infections, although their inclusion reduced the magnitude and consistency of the alpha-diversity reductions.

## 4. Discussion

The COVID-19 pandemic provided a rare natural experiment in which transmission networks were disrupted on a national and global scale. Our results identify temporal changes in within-host *gp60* diversity and ASV composition that are consistent with altered transmission conditions during and after the COVID-19 pandemic and suggest that such disruptions may be associated with measurable genetic signatures within parasite populations and within individual infections. More broadly, our findings highlight the value of within-host diversity as an indicator of transmission processes and the magnitude and consistency of temporal changes according to transmission ecology.

The reduction in *C. hominis* diversity observed here suggests that within-host genetic complexity may reflect recent changes in transmission history. Post-COVID *C. hominis* infections contained fewer *gp60* variants and lower within-host diversity, accompanied by a shift in *gp60* ASV composition. Together, these findings are consistent with a possible reduction in effective parasite connectivity resulting from the interruption of predominantly human-associated transmission pathways [21,22]. Reduced opportunities for human-associated transmission, through altered contact, mobility, environmental contamination and exposure pathways, may have limited the circulation and mixing of parasite lineages, decreasing the probability that genetically diverse infections would be generated and maintained.

Relative-abundance Bray–Curtis dispersion was lower after COVID, indicating that non-outbreak post-COVID *C. hominis* infections were more compositionally homogeneous. This suggests a contraction in the diversity of circulating variants, with individual infections harbouring fewer ASVs and showing increased dominance by one or a few variants, a pattern consistent with reduced exposure to genetically diverse transmission sources. Because the Bray–Curtis PERMANOVA was accompanied by a significant dispersion difference, it cannot be interpreted solely as evidence of a directional shift in group centroids. Nevertheless, the binary Jaccard analysis detected a significant temporal difference without a corresponding change in dispersion, providing stronger evidence that the set of ASVs occurring within *C. hominis* infections changed between periods. The absence of dispersion differences indicates that the significant Jaccard PERMANOVA reflects a directional change in variant occurrence rather than increased heterogeneity among infections. Together with the reductions in alpha diversity and ASV richness, these findings are consistent with a reduction in the diversity and complexity of parasite variants detected in predominantly human-associated transmission pathways. Such a pattern would be compatible with reduced transmission connectivity and fewer opportunities for hosts to acquire multiple parasite lineages, although demographic bottlenecks and gene flow were not estimated directly [1,2]. Direct evidence of altered gene flow would require multilocus or whole-genome data and population-genetic inference.

*Cryptosporidium parvum* provides further support for a transmission-network interpretation, displaying a different response. Although significant differences in beta diversity were detected, the absence of corresponding reductions in within-host diversity suggests that parasite connectivity was not disrupted to the same extent. The observed changes in beta diversity coincided with significant differences in dispersion but were not accompanied by reductions in alpha diversity. Together, these findings indicate that post-COVID *C. parvum* infections became more compositionally homogeneous rather than more heterogeneous. *Cryptosporidium parvum* exhibited differences in multivariate composition; however, because these coincided with significant differences in dispersion, the observed PERMANOVA probably reflects changes in heterogeneity among infections rather than clear directional turnover in variant composition. Because significant temporal PERMANOVA differences were accompanied by significant differences in dispersion for both Bray–Curtis and Jaccard distances, the evidence for directional restructuring of ASV composition is less clear than for *C. hominis*. Nevertheless, the absence of reductions in Shannon, Simpson and inverse Simpson diversity or ASV richness is consistent with the possibility that animal and environmental sources may buffer *C. parvum* against a major loss of within-host diversity or reorganization of the relative abundance structure of circulating variants without a corresponding loss of overall richness. Livestock and environmental reservoirs may therefore have continued to sustain parasite transmission despite restrictions on human movement and social contact. Similarly, the lack of a signal in *C. cuniculus* may reflect continued maintenance within animal reservoirs and associated environmental transmission pathways. No significant temporal difference was detected in Shannon diversity, Simpson diversity, ASV richness, Bray–Curtis composition or Jaccard composition for *C. cuniculus*. Inverse Simpson diversity was significantly lower post-COVID, but this isolated result was not consistently supported by the other alpha-diversity measures. Given the limited sample size of 12 infections per period, the findings for this species should be regarded as descriptive rather than inferential, and additional sampling will be required to determine whether these observations are genuine.

Although our study was not designed to estimate effective population size or quantify formal bottleneck parameters, the observed reduction in within-host diversity is consistent with expectations under transmission limitation. Future studies incorporating whole-genome sequencing, longitudinal sampling and coalescent-based demographic inference could test these hypotheses directly and determine whether the diversity losses observed in *C. hominis* represent transient fluctuations or longer-term within-host variant composition. From an evolutionary perspective, the results are consistent with the concept that anthropogenic changes in host connectivity and environmentally mediated exposure pathways may rapidly influence parasite population structure [15,20,48]. The time scale over which these changes occurred is striking and demonstrates the responsiveness of parasite populations to shifts in transmission opportunity. These responses may have implications for adaptive potential, lineage persistence and epidemic dynamics. Because *gp60* is under immune selection and exhibits extensive subtype diversity, reductions in within-host variant richness may provide a sensitive indicator of recent changes in transmission [34,49–52].

In conclusion, the period following the onset of the COVID-19 pandemic left a detectable signature in the genetic diversity of *Cryptosporidium*. The selective reduction of diversity in the anthroponotic species, but not in predominantly zoonotic species, is consistent with a central role of human-associated transmission, including both person-to-person and environmentally mediated pathways originating from human infections. These findings are consistent with changes in human behaviour, mobility and exposure pathways rapidly altering parasite connectivity and the circulation of parasite variants and thus gene flow over just a few transmission generations. This illustrates how quickly ecological change can reshape parasite genetic diversity and highlights the value of within-host metabarcoding for detecting contemporary evolutionary change.

## Supplementary Material



## Data Availability

Raw sequence reads are deposited in the NCBI Sequence Read Archive under BioProject accession PRJNA1507456. Code is available at Zenodo [53]. Supplementary material is available online [54].
