# Supplementary material for: Contrasting pre- and post-COVID changes in within-host gp60 diversity among human-infecting Cryptosporidium species
Source: R Soc Open Sci. 2026 Nov 11;13(11):261952. doi: 10.1098/rsos.261952 (PMC13750924; doi:10.1098/rsos.261952)
Supplement: Supplementary file 1 [file rsos.261952.zip › rsos.261952/rsos261952_si_001.docx]

**Supplementary Methods and Results**

Table S1. Shannon alpha-diversity estimates based on the complete dataset, including *Cryptosporidium hominis* samples collected during the 2023 Queenstown outbreak.

| **Species** | **estimate** | **SE** | **df** | **lower.CL** | **upper.CL** | **t.ratio** | **p.value** |
| --- | --- | --- | --- | --- | --- | --- | --- |
| ***C. hominis*** | -0.4495 | 0.14 | 326 | -0.724 | -0.175 | -3.219 | 0.0014 |
| ***C. parvum*** | -0.0813 | 0.104 | 326 | -0.286 | 0.124 | -0.781 | 0.4354 |
| ***C. cuniculus*** | -0.436 | 0.274 | 326 | -0.974 | 0.102 | -1.594 | 0.1119 |

Table S2. Simpson alpha-diversity estimates based on the complete dataset, including *Cryptosporidium hominis* samples collected during the 2023 Queenstown outbreak.

| **Species** | **estimate** | **SE** | **df** | **lower.CL** | **upper.CL** | **t.ratio** | **p.value** |
| --- | --- | --- | --- | --- | --- | --- | --- |
| ***C. hominis*** | -0.08905 | 0.0359 | 326 | -0.1597 | -0.0184 | -2.479 | 0.0137 |
| ***C. parvum*** | -0.00998 | 0.0268 | 326 | -0.0627 | 0.0427 | -0.373 | 0.7097 |
| ***C. cuniculus*** | -0.0543 | 0.0704 | 326 | -0.1927 | 0.0841 | -0.772 | 0.4408 |

Table S3. ASV richness estimates based on the complete dataset, including *Cryptosporidium hominis* samples collected during the 2023 Queenstown outbreak.

| **Species** | **ratio** | **SE** | **df** | **asymp.LCL** | **asymp.UCL** | **null** | **z.ratio** | **p.value** |
| --- | --- | --- | --- | --- | --- | --- | --- | --- |
| ***C. hominis*** | 0.530 | 0.0654 | Inf | 0.416 | 0.675 | 1 | -5.143 | <0.0001 |
| ***C. parvum*** | 0.908 | 0.0801 | Inf | 0.764 | 1.08 | 1 | -1.092 | 0.275 |
| ***C. cuniculus*** | 0.713 | 0.165 | Inf | 0.453 | 1.122 | 1 | -1.461 | 0.144 |

Table S4. Species-specific beta-diversity analyses including *Cryptosporidium hominis* samples collected during the 2023 Queenstown outbreak.

| **Species** | **N** | **N (Pre)** | **N (Post)** | **R2** | **F** | **PERMANOVA_p** | **Pre_mean_dist_**  **to_centroid** | **Post_mean_dist_**  **to_centroid** | **Dispersion_p** |
| --- | --- | --- | --- | --- | --- | --- | --- | --- | --- |
| ***C. hominis*** | 142 | 100 | 42 | 0.199 | 34.706 | 0.001 | 0.553 | 0.504 | 0.205 |
| ***C. parvum*** | 167 | 78 | 89 | 0.098 | 17.958 | 0.001 | 0.580 | 0.485 | 0.019 |
| ***C. cuniculus*** | 24 | 12 | 12 | 0.040 | 0.928 | 0.494 | 0.616 | 0.627 | 0.822 |

Figure S1. Comparison of within-host diversity between pre- and post-COVID samples. (A) Shannon diversity, (B) Simpson diversity, (C) Inverse Simpson, and (D) Amplicon Sequence Variant (ASV) richness for *Cryptosporidium hominis* (including outbreak samples from 2023 in Queenstown, New Zealand), *C. parvum*, and *C. cuniculus*. Boxes show the interquartile range with the median; whiskers represent 1.5 × IQR, and points represent individual samples.

Figure S2. Principal coordinates analysis (PCoA) based on Bray–Curtis dissimilarity of gp60 amplicon sequence variants. Samples are coloured according to the sampling period (pre- and post-COVID). Separation of centroids was assessed using PERMANOVA, and differences in multivariate dispersion were evaluated using tests of homogeneity of multivariate dispersion (betadisper). Panels correspond to *Cryptosporidium hominis* (including outbreak samples from 2023 in Queenstown, New Zealand), *C. parvum*, and *C. cuniculus*.
